# Varieties of semantic ‘access’ deficit in Wernicke’s aphasia and semantic aphasia

**DOI:** 10.1093/brain/awv281

**Published:** 2015-10-10

**Authors:** Hannah E. Thompson, Holly Robson, Matthew A. Lambon Ralph, Elizabeth Jefferies

**Affiliations:** 1 Department of Psychology and York Neuroimaging Centre, University of York, UK; 2 School of Psychology and Clinical Language Sciences, University of Reading, UK; 3 Neuroscience and Aphasia Research Unit, School of Psychological Sciences, University of Manchester, UK

**Keywords:** semantic, Wernicke, aphasia, modality, refractory

## Abstract

Comprehension deficits are common in stroke aphasia, including in cases with (i) semantic aphasia, characterized by poor executive control of semantic processing across verbal and non-verbal modalities; and (ii) Wernicke’s aphasia, associated with poor auditory–verbal comprehension and repetition, plus fluent speech with jargon. However, the varieties of these comprehension problems, and their underlying causes, are not well understood. Both patient groups exhibit some type of semantic ‘access’ deficit, as opposed to the ‘storage’ deficits observed in semantic dementia. Nevertheless, existing descriptions suggest that these patients might have different varieties of ‘access’ impairment—related to difficulty resolving competition (in semantic aphasia) versus initial activation of concepts from sensory inputs (in Wernicke’s aphasia). We used a case series design to compare patients with Wernicke’s aphasia and those with semantic aphasia on Warrington’s paradigmatic assessment of semantic ‘access’ deficits. In these verbal and non-verbal matching tasks, a small set of semantically-related items are repeatedly presented over several cycles so that the target on one trial becomes a distractor on another (building up interference and eliciting semantic ‘blocking’ effects). Patients with Wernicke’s aphasia and semantic aphasia were distinguished according to lesion location in the temporal cortex, but in each group, some individuals had additional prefrontal damage. Both of these aspects of lesion variability—one that mapped onto classical ‘syndromes’ and one that did not—predicted aspects of the semantic ‘access’ deficit. Both semantic aphasia and Wernicke’s aphasia cases showed multimodal semantic impairment, although as expected, the Wernicke’s aphasia group showed greater deficits on auditory-verbal than picture judgements. Distribution of damage in the temporal lobe was crucial for predicting the initially ‘beneficial’ effects of stimulus repetition: cases with Wernicke’s aphasia showed initial improvement with repetition of words and pictures, while in semantic aphasia, semantic access was initially good but declined in the face of competition from previous targets. Prefrontal damage predicted the ‘harmful’ effects of repetition: the ability to reselect both word and picture targets in the face of mounting competition was linked to left prefrontal damage in both groups. Therefore, patients with semantic aphasia and Wernicke’s aphasia have partially distinct impairment of semantic ‘access’ but, across these syndromes, prefrontal lesions produce declining comprehension with repetition in both verbal and non-verbal tasks.

## Abstract (precis)

Comprehension deficits are common in both Wernicke’s aphasia and semantic aphasia. Thompson *et al.* compare these groups of patients on a task in which related items are repeatedly presented, increasing semantic competition across repetitions. Both groups show semantic impairment, but only individuals with prefrontal damage show harmful effects of repetition.

## Graphical abstract

**Figure:**
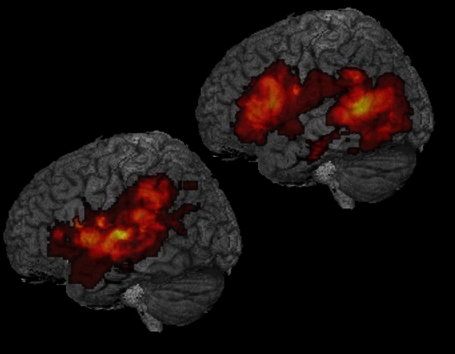
Comprehension deficits are common in both Wernicke’s aphasia and semantic aphasia. Thompson *et al.* compare these groups of patients on a task in which related items are repeatedly presented, increasing semantic competition across repetitions. Both groups show semantic impairment, but only individuals with prefrontal damage show harmful effects of repetition.

## Introduction

Semantic cognition allows us to understand the significance of words and objects that we encounter. It encompasses several interacting components within a widely distributed neural network (Patterson *et al.*, 2007; Jefferies, 2013), giving rise to different types of semantic impairment after brain damage (Jefferies and Lambon Ralph, 2006; Robson *et al.*, 2012). Progressive degradation of conceptual representations is observed in semantic dementia following atrophy of the anterior temporal lobes (Snowden *et al.*, 1989; Hodges *et al.*, 1992; Mummery *et al.*, 2000; Galton *et al.*, 2001). In other patients, knowledge is preserved but deficient semantic ‘access’ prevents the effective retrieval of this information (Lambon Ralph, 2014). Patients can show difficulties mapping sensory perception into semantic knowledge, such as the disordered translation of auditory input into semantics in word deafness and Wernicke’s aphasia (Goldblum and Albert, 1972; Tanaka *et al.*, 1987; Poeppel, 2001; Slevc *et al.*, 2011; Robson *et al.*, 2013). Further, there may be a deficit of controlled selection/retrieval of semantic knowledge that is sensitive to task demands, as in semantic aphasia (Jefferies and Lambon Ralph, 2006).

Wernicke’s aphasia is often thought of as the ‘quintessential’ comprehension disorder in stroke aphasia. It is characterized by impaired auditory comprehension and repetition, paired with fluent speech involving phonological paraphasias and neologisms (Goodglass *et al.*, 2001*a*). Other linguistic skills such as reading and writing can be relatively preserved (Ellis *et al.*, 1983), suggesting there is a continuum in Wernicke’s aphasia of auditory linguistic and non-auditory abilities which are partially dissociable (Robson *et al.*, 2012). Because of this, Wernicke’s aphasia is commonly considered to be based on an auditory ‘input’ or phonological perception deficit (Harris, 1970; Luria, 1970; Goldblum and Albert, 1972). However, this account is challenged by the observation that other aphasic patients can be equally impaired at phonemic discrimination tasks without showing severe semantic access deficits at the single word level (Blumstein *et al.*, 1977). Moreover, patients with Wernicke’s aphasia can exhibit impairment on non-verbal as well as verbal semantic tasks, although they are notably worse at understanding spoken words (De Renzi *et al.*, 1972; Cohen *et al.*, 1980; Gainotti *et al.*, 1983; Ogar *et al.*, 2011). For example, recent evidence suggests that patients with Wernicke’s aphasia can have additional multimodal semantic control impairments (Robson *et al.*, 2012).

Although auditory–verbal comprehension in Wernicke’s aphasia has been most commonly studied, a long parallel literature has examined stroke patients with ‘multimodal’ semantic deficits (semantic aphasia; Head, 1926; Luria, 1973). Luria (1970) described patients who were unable to integrate relationships between concepts, while Head (1926) characterized semantic aphasia as a deficit in comprehending the full extent of meaning of words and pictures. Both descriptions emphasize a deficit of complex semantic processing across modalities, with difficulty drawing inferences beyond dominant or literal interpretations (see also Hier *et al.*, 1980; Ardila *et al.*, 2000). We have used the term ‘semantic aphasia’ to refer to a multimodal semantic deficit in which there is difficulty in the controlled ‘application’ of knowledge correlating with executive impairment (Jefferies and Lambon Ralph, 2006; Jefferies *et al.*, 2007; Corbett *et al.*, 2009*b*; Hoffman *et al.*, 2010; Noonan *et al.*, 2010; Jefferies, 2013; Lambon Ralph, 2014). These features share some similarities with the original descriptions of semantic aphasia (for review, see Gainotti, 2014) despite researchers emphasizing different characteristics and using different tasks. The original definitions of semantic aphasia provided by Head (1926) and Luria (1973) referred to a ‘high-level’ deficit in understanding multiple concepts with mild (if any) deficits in single object processing. In this study, and previous publications by the current authors, the majority of semantic aphasia cases had large frontoparietal lesions and comprehension problems at a single-item level, though the patients’ impairments were still more apparent with more complex stimuli. The term ‘semantic aphasia’ transcends classical ‘Boston’ aphasia classifications; however, many patients with semantic aphasia present with the profile of transcortical sensory aphasia, displaying good repetition—at least no worse than would be expected from spontaneous speech production—and speech free from jargon. Cases with semantic aphasia show deregulated verbal and non-verbal semantic behaviour, especially when they are required to use conceptual information in a flexible way, in the absence of few external constraints (Corbett *et al.*, 2009*a*, *b*, 2011). Their semantic performance is (i) relatively good on tasks with minimal control demands, such as matching words or objects that are highly associated (e.g. ‘salt’ with ‘pepper’), but poorer for weak associations (e.g. ‘salt’ with ‘sugar’; Noonan *et al.*, 2010); (ii) highly consistent across decisions involving words and pictures, but not across tasks with different executive demands, such as word-picture matching and association judgements (Jefferies and Lambon Ralph, 2006); and (iii) susceptible to being aided by cues and misled by miscues that are designed to activate the target or distracters in both picture naming and object use demonstrations (Jefferies *et al.*, 2007; Soni *et al.*, 2009; Noonan *et al.*, 2010; Corbett *et al.*, 2011).

Current descriptions of Wernicke’s aphasia and semantic aphasia suggest these groups may show somewhat distinct, yet partially overlapping deficits of semantic ‘access’. These problems have been previously explored (and partly defined by) the ‘classical’ paradigm of cyclical word–picture matching (Warrington and McCarthy, 1983; Forde and Humphreys, 1997; Warrington and Crutch, 2004). In semantic ‘access’ patients, including cases with semantic aphasia, accuracy declines when a small set of semantically-related items are repeatedly tested over a number of cycles, with the target on one trial becoming a distractor on the next (Forde and Humphreys, 1997; Warrington and Crutch, 2004; Jefferies *et al.*, 2007; Gardner *et al.*, 2012; Thompson and Jefferies, 2013). The same effect has been shown in non-repeating related items across a session (Warrington and McCarthy, 1983; Forde and Humphreys, 1997), suggesting that competition builds up between semantic associates making it increasingly difficult to select the appropriate target. However, competition in the traditional cyclical task is particularly strong since it requires: (i) inhibition of items after they have been selected; and (ii) reselection when these items subsequently become targets again in later cycles (Thompson-Schill *et al.*, 1997; Gotts and Plaut, 2002; Badre *et al.*, 2005; Jefferies *et al.*, 2007; Mirman *et al.*, 2013).

Even though cyclical word–picture matching tasks have been paradigmatic in establishing the existence of semantic ‘access’ impairment, research using these tasks has typically examined single cases or small clusters of patients selected on the basis that they show declining performance over cycles (Warrington and McCarthy, 1983; Warrington and Cipolotti, 1996; Forde and Humphreys, 1997; Warrington and Crutch, 2004; Crutch and Warrington, 2008; Thompson and Jefferies, 2013). As a consequence, not enough is known about the typical profile of comprehension deficits following stroke, and whether declining semantic ‘access’ with repetition is a common problem—yet this issue has important clinical and theoretical implications. This study tackled these questions by comparing the comprehension of two groups (Wernicke’s aphasia and semantic aphasia), with overlapping yet distinct aphasia and lesion profiles, on classical cyclical spoken word-picture and picture-picture matching for the first time. The patients were not specifically selected to show effects of cycle—instead, this was our key outcome measure. We used a comparative case series approach (Lambon Ralph *et al.*, 2007, 2011) to investigate group-level differences and similarities, as well as the causes of individual variation within the groups, examining the impact of patient group (semantic aphasia versus Wernicke’s aphasia), modality (pictures versus words), lesion location (prefrontal versus temporoparietal lesions) and item repetition (charting improvement or decline in comprehension as concepts are repeated). Below, each of these variables is discussed in more detail.

### Patient group

Clinical labels are important in aphasia research and therapy as they capture meaningful constellations of symptoms and make predictions about how individuals will perform in multiple tasks (Henseler *et al.*, 2014); however, these classifications are graded rather than absolute, can be partially overlapping and different types of patient can show similar deficits on specific tasks (Butler *et al.*, 2014). Deficient executive control over semantic processing is a core feature of semantic aphasia but has recently been extended to predict behaviour in Wernicke’s aphasia (Robson *et al.*, 2012). Thus, we might anticipate overlapping deficits in patients with semantic aphasia and Wernicke’s aphasia.

### Lesion location

Classifications of aphasic symptoms were devised before neuroimaging methods provided us with detailed information about the functions of specific regions of cortex. These insights suggest that aphasic symptoms should be strongly predicted by lesion location; nonetheless, individual circumstances (e.g. pre-stroke anatomical functioning, age at stroke, amount of therapy post-stroke etc.) affect functional adjustment to brain injury, meaning that lesion location is not deterministic but affects the probability of particular impairments. On average temporoparietal damage is more extrasylvian than perisylvian in semantic aphasia than Wernicke’s aphasia (Chertkow *et al.*, 1997; Berthier, 1999; Dronkers *et al.*, 2004; Robson *et al.*, 2012). Wernicke’s aphasia is particularly associated with damage to the superior temporal gyrus (Eggert, 1977), an area linked to speech perception at a phonological level (Hickok and Poeppel, 2000; Buchsbaum *et al.*, 2001; Okada and Hickok, 2006) and damage here is also thought to account for phonemic paraphasias and poor repetition/naming in Wernicke’s aphasia (Damasio, 1992; Goodglass, 1992). In contrast, the temporal lobe damage in semantic aphasia is focused on posterior middle and inferior temporal gyrus (Berthier, 1999; Noonan *et al.*, 2010; Corbett *et al.*, 2011; Jefferies, 2013), brain areas linked to both word and picture-based semantic tasks.

Individuals in both groups can have left inferior frontal gyrus damage (Gardner *et al.*, 2012; Robson *et al.*, 2012), and this is not predictive of aphasia classification yet could relate to semantic access deficits. Previous work has suggested that patients with damage to left inferior frontal gyrus show declining semantic performance over cycles, whereas lesions restricted to posterior temporal cortex do not elicit this pattern (Jefferies *et al.*, 2007; Campanella *et al.*, 2009; Schnur *et al.*, 2009; Gardner *et al.*, 2012). These findings are broadly consistent with the proposal that left inferior frontal gyrus is crucial for post-retrieval selection (Badre *et al.*, 2005; Badre and Wagner, 2007), particularly when a concept has been activated and inhibited, and then needs to be reselected. Some researchers have explicitly linked left inferior frontal gyrus damage to lexical selection ‘for speech production’ and have suggested that patients do not show the same pattern in comprehension tasks (Schnur *et al.*, 2006, 2009). The current study examines if this relationship with lesion location holds, irrespective of patient classification for semantic matching tasks.

### Modality

Much of the previous work on semantic ‘access’ deficits has targeted the auditory–verbal domain (Warrington and McCarthy, 1983, 1987; McNeil *et al.*, 1994; Warrington and Cipolotti, 1996; Jefferies *et al.*, 2007). Recently, however, comparisons of verbal and non-verbal cyclical matching tasks have become a focus of debate: some individual patients appear to have an ‘access’ impairment that is limited to verbal tasks (Warrington and McCarthy, 1983; Warrington and Cipolotti, 1996; Warrington and Crutch, 2004; Crutch and Warrington, 2008), while we and others have proposed that semantic access/control mechanisms are domain-general and occur across modalities (Forde and Humphreys, 1997; Corbett *et al.*, 2009*a*, 2011; Gardner *et al.*, 2012). It is perhaps unsurprising that cases with semantic aphasia show a decline in comprehension for both words and pictures on cyclical matching tasks, since they have multimodal semantic control deficits (although the patients are not specifically recruited to show deficits in executive-semantic processing or effects of repetition/cycle). In contrast, as cases with Wernicke’s aphasia typically show poorer comprehension of spoken words than pictures, it might be that these patients have marked access deficits in the verbal domain and, as a consequence, resemble the semantic ‘access’ patients studied by Warrington and colleagues (like the single case described by Thompson and Jefferies, 2013).

### Repetition

Semantic ‘access’ patients show declining performance when stimuli are repeated in the cyclical matching paradigm even though repetition in other tasks typically enhances performance. When errors arise from a failure to ‘activate’ concepts from a particular sensory input (Dell *et al.*, 1997), repetition should ameliorate this deficit. Wernicke’s aphasics show strong facilitation with repetition priming, i.e. when a target is presented that is identical to a prime (Blumstein *et al.*, 2000). However, patients with Wernicke’s aphasia have also shown interference effects and can be negatively affected by repetition through activation of related distractors—just like patients with semantic aphasia. For example, patients with Wernicke’s aphasia can show impairment on tasks that have phonemically overlapping primes (e.g. ‘piano-pyjamas’) or phonologically related distractors (e.g. hammer with ‘hammock’), suggesting a difficulty in deactivating competing word candidates (Weiner *et al.*, 2004; Janse, 2006; Yee *et al.*, 2008). Indeed, Mirman and colleagues (2013) suggested that impaired input processing (such as that observed in Wernicke’s aphasia) makes residual activation of previously processed items more difficult for the new input to overcome. Therefore, patients with Wernicke’s aphasia may show a mixture of both facilitation from repetition (due to failure to activate concepts initially), followed by interference from overactivation of competing concepts (Campanella and Shallice, 2011). Patients with semantic aphasia, on the other hand, are unlikely to show initial facilitation from repetition, due to intact input processing mechanisms.

In summary, although patients with semantic aphasia and Wernicke’s aphasia are considered to have problems ‘accessing’ conceptual representations, rather than degradation of semantic representations *per se* (e.g. as observed in semantic dementia: Warrington and Cipolotti, 1996; Jefferies and Lambon Ralph, 2006), these groups have not been directly compared on cyclical matching tasks designed to elicit these deficits. This study establishes (i) whether semantic ‘access’ impairment is a common problem for comprehension-impaired people with stroke aphasia (in cases not specifically selected to show the pattern of declining accuracy over repeated cycles); (ii) whether Wernicke’s aphasia and semantic aphasia cases show qualitatively the same type of semantic access disorder; (iii) to what extent access disorders are limited to the verbal domain (in either group); and (iv) what accounts for variability ‘within’ semantic aphasia and Wernicke’s aphasia, assessing effects of lesion location, modality and repetition.

## Materials and methods

### Participants

#### Semantic aphasia

Aphasia profiles and demographic information are displayed in Table 1. Thirteen patients with semantic aphasia took part in this experiment. Data are included from nine patients with semantic aphasia reported by Gardner *et al.* (2012), along with four additional patients. In line with previous studies on semantic aphasia, patients were selected on the basis of semantic comprehension deficits affecting both words and pictures using the Camel and Cactus task of semantic association (Bozeat *et al.*, 2000). They were not specifically selected to show effects of cycle in matching tasks or deficient semantic control. While some of the patients had relatively selective comprehension deficits (and thus presented with a pattern of ‘transcortical sensory aphasia’), speech fluency and repetition scores were not used as selection criteria and therefore patients within the group had variable language deficits affecting these domains. All patients had chronic impairment after a cerebrovascular accident at least 1 year before testing.

**Table 1:** Aphasia profiles and demographic information for patients with semantic aphasia and Wernicke’s aphasia

Case	Age	Sex	Full-time education (leaving age)	Aphasia classification	BDAE fluency percentile	Repetition		Cambridge comprehension – spoken	BDAE comprehension percentile
						Non-words (% correct)	Words (% correct)		
HN	80	M	15	Anomic/TSA	NT	56	86	56	NT
SC	76	M	16	Anomic/TSA	90	87	98	89	37
ME	36	F	16	TSA	100	93	100	81	33
KS	59	M	16	TSA	97	73	94	72	43
EW	74	F	15	TSA	NT	NT	80	91	NT
PG	59	M	18	TSA	40	73	91	94	20
NY	63	M	15	Mixed transcortical	37	40	81	89	47
BB	55	F	16	Mixed transcortical	17	83	96	76	10
DB	83	M	16	TSA	90	70	85	73	13
GH	55	F	15	Global	NT^a^	NT^a^	16^a^	94	NT
EC	M	70	16	Global	NT^a^	0^a^	0^a^	63	NT
KA	74	M	14	Global	23^a^	0^a^	0^a^	49	0
LS	71	M	15	TSA	90	90	96	74	13
EL	62	M	15	WA	96	0	18	45	14
MR	66	M	15	WA	83	4	8	52	20
CW	71	M	15	WA	91	13	49	71	48
DMC	68	M	18	WA	80	NT	0	25	10
DR	77	M	15	WA	74	NT	1	14	5
LaS	67	M	15	WA	85	NT	6	50	15
DL	74	M	15	WA	90	NT	1	13	8
CB	61	M	15	WA	38	NT	4	NT	10

^a^Low fluency with minimal words produced on a cookie theft task (Goodglass and Kaplan, 1983).

BDAE = Boston Diagnostic Aphasia Examination (Goodglass and Kaplan, 1983).

BDAE Comprehension percentile is derived from three subtests (word discrimination, commands, complex ideational material). Cambridge comprehension refers to an average percentage score on spoken word-to-picture matching tasks found in the Cambridge Semantic Battery (Bozeat *et al.*, 2000) and the environmental sounds task (Bozeat *et al.*, 2003). BDAE fluency percentile is derived from phrase length, melodic line and grammatical form ratings. BDAE Repetition percentile is an average of word and sentence repetition subtests. Word/non-word repetition = Tests 8 and 9 from Psycholinguistic Assessments of Language Processing in Aphasia: PALPA (Kay et al., 1992). Aphasia classifications were based on fluency, repetition and comprehension. TSA (transcortical sensory aphasia) was defined as good or intermediate fluency/repetition and poorer comprehension. Wernicke’s aphasia (WA) was defined as relatively fluent speech with poor repetition and comprehension. NT = not tested.

#### Wernicke’s aphasia

Eight patients with Wernicke’s aphasia were selected after a single left hemisphere stroke to show classic symptoms of Wernicke’s aphasia: a single word comprehension impairment, fluent sentence-like speech punctuated with phonological or neologistic errors, and errors on single word repetition and naming. Participants were screened using the Boston Diagnostic Aphasia Examination (Goodglass *et al.*, 2001*b*) to show comprehension impairment below the 45th percentile and sentence repetition impairment below the 65th percentile. The average phrase length in everyday speech was required to be above six words. In structured picture description, paraphasias had to occur every few utterances as a minimum. These were largely phonological (e.g. ‘papple’ for apple) or neologistic.

#### Patient lesion analysis

**Figure 1:**
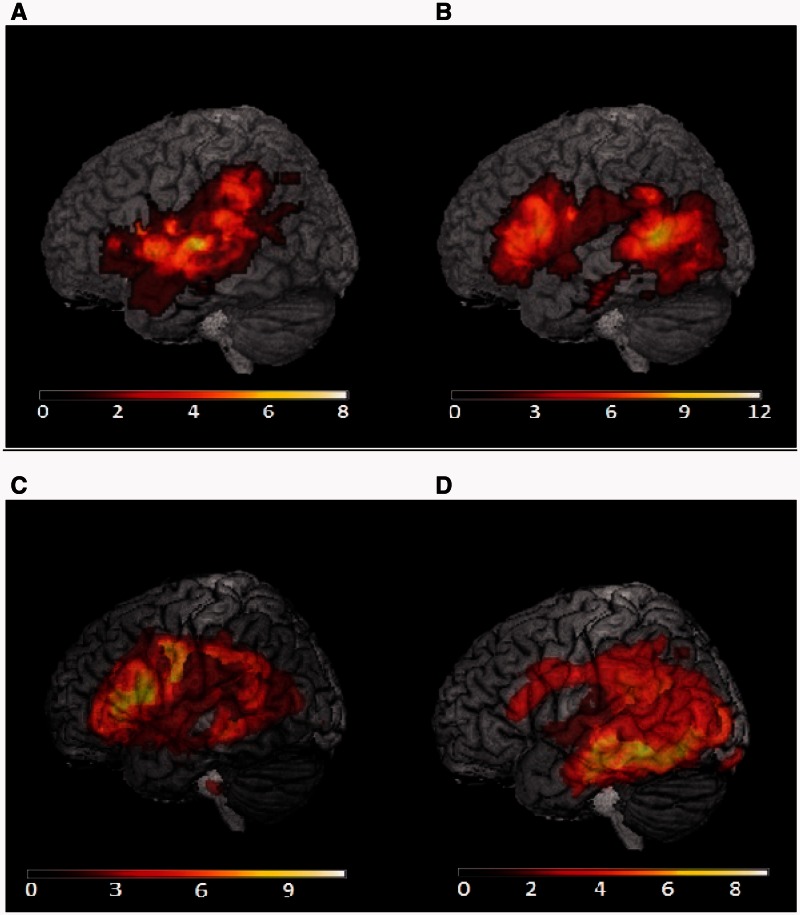
**Lesion overlay map from automatic lesion identification.** Lightest colours show areas of maximal overlap between subjects. There were eight patients with Wernicke’s aphasia, and 12 patients with semantic aphasia included in this lesion analysis (shown in **A** and **B**; a further semantic aphasia patient was not scanned). In the prefrontal group (**C**), there were four patients with Wernicke’s aphasia and eight patients with semantic aphasia (one semantic aphasia patient with a prefrontal cortex lesion was not scanned). In the temporoparietal group, there were four patients with Wernicke’s aphasia and five with semantic aphasia (**D**). To produce these images, the patients’ brains were compared to aged-matched control MRI scans, which were collected at the Universities of Manchester and York. Grey matter, white matter and CSF were segmented and changes from the healthy control brains were highlighted as ‘lesion’ (or more precisely, an unexpected tissue class) using automated methods (Seghier *et al.*, 2008). For the Manchester patients there were 19 controls, with a mean age of 68.2 years [standard deviation (SD) = 5.99; eight female, 11 male]. For the York patients, there were 14 controls, mean age of 64.7 years (SD = 6.5, eight female, six male). The automatic lesion identification algorithm was run separately for Manchester and York cases who were examined using a different MRI scanner. CT scans (Patients MR, BB and KA) were hand-drawn onto a template (Damasio and Damasio, 1989), allowing these patients to be included in these images.

CT/MRI scans were available for all eight patients with Wernicke’s aphasia (one CT scan, seven MRI scans), and 12/13 patients with semantic aphasia (two CT scans, 10 MRI scans). A scan was not available for one semantic aphasia case (Patient PG) due to contraindications for MRI; a report of a CT scan in the acute phase indicated a left frontal lesion. An overlay of lesion maps created from automated lesion identification (Seghier *et al.*, 2008) is displayed in Fig. 1.

Table 2 displays details of the patients’ lesions, focusing on regions of interest in temporal, parietal and frontal cortex. The inferior anterior temporal lobe, implicated in amodal semantic representation and atrophied in semantic dementia, was spared in all cases (Binney *et al.*, 2010; Mion *et al.*, 2010). The main area of lesion overlap in semantic aphasia was in posterior middle temporal and inferior temporal gyrus [Brodmann area (BA) 37 and BA21]. In the Wernicke’s aphasia group, there was greater damage to the posterior superior temporal and supramarginal gyri (BA 22 and BA 40). Nearly all patients with Wernicke’s aphasia had damage to anterior-to-mid superior temporal gyrus, where almost none of the semantic aphasia cases did. Similarly, the posterior occipital-temporal region was nearly always damaged in semantic aphasia patients but not in those with Wernicke’s aphasia. The groups could be entirely separated on the basis of temporal lobe lesion location after the removal of a few cases (Patient EL from the Wernicke’s aphasia group and Patients BB, GH and EC from the semantic aphasia group). Thus, the distribution of damage in the temporal lobe was a key predictor of aphasia classification (Supplementary material and Supplementary Tables 1 and 2).

**Table 2:** Details of semantic aphasia and Wernicke’s aphasia patients’ lesions

			Prefrontal						Anterior temporal		Posterior temporal					Parietal	
**Patient**	Lesion group	Lesion size (%)^a^		DLPFC	orbIFG		trIFG	opIFG	sTP	aSTG	pSTG	pMTG	pITG	FG	POT	AG	SMG
			BA9	BA46	BA47		BA45	BA44	BA38	BA22	BA22	BA21	BA20	BA36	BA37	BA39	BA40
**HN**	SA TP-only	6	-	-	-		-	-	-	-	-	2	1	-	2	-	-
**SC**	SA TP-only	8	-	-	-		-	-	-	-	-	2	2	-	2	2	1
**ME**	SA TP-only	5	-	-	-		-	-	-	-	-	1	2	2	1	-	-
**KS**	SA TP-only	2	-	-	-		-	-	-	-	1	2	-	-	2	-	-
**EW**	SA TP-only	2	-	-	-		-	-	-	-	-	-	1	-	1	-	-
**NY**	SA PF+	14	-	1	2		2	2	-	-	2	-	-	-	-	1	1
**BB**	SA PF+	3	-	-	2		2	2	-	1	1	-	-	-	-	-	-
**DB**	SA PF+	12	1	1	1		2	2	-	-	2	1	-	-	-	-	1
**GH**	SA PF+	12	-	-	2		1	1	-	1	2	1	-	-	2	1	1
**EC**	SA PF+	17	-	-	2		1	2	1	1	2	1	-	-	1	-	-
**KA**	SA PF+	6	-	-	-		-	2	-	-	2	1	-	-	1	-	1
**LS**	SA PF+	17	-	1	-		2	2	-	-	-	2	2	-	2	2	2
**% SA patients with damage to that region**			8	25	42		50	58	8	25	58	75	42	8	75	33	50
**EL**	WA TP-only	5	-	-	-		-	-	-	-	1	-	-	-	-	1	2
**MR**	WA TP-only	3	-	-	-		-	-	-	1	2	1	-	-	-	-	2
**CW**	WA TP-only	4	-	-	-		-	-	-	1	2	-	-	-	-	-	2
**DMC**	WA TP-only	16	-	-	-		-	-	1	1	2	1	-	-	-	2	2
**DR**	WA PF+	8	1	-	-		2	2	-	1	1	-	-	-	-	1	1
**LaS**	WA PF+	12	-	-	1		-	2	1	2	2	2	-	-	1	1	2
**DL**	WA PF+	8	-	-	-		2	1	2	2	1	2	-	-	-	-	2
**CB**	WA PF+	17	-	-	1		2	2	1	2	2	-	-	-	-	-	2
**% WA patients with damage to that region**			13	0	25		38	50	50	88	100	50	0	0	13	50	100

Quantification of lesion: 2 = complete destruction/serious damage to cortical grey matter; 1 = partial destruction/mild damage to cortical grey matter. Anatomical abbreviations: DLPFC = dorsolateral prefrontal cortex; orbIFG = pars orbitalis in inferior frontal gyrus; trIFG = pars triangularis in inferior frontal gyrus; opIFG = pars opercularis in inferior frontal gyrus; sTP = superior temporal pole; STG = superior temporal gyrus; MTG = middle temporal gyrus; ITG = inferior temporal gyrus; FG = fusiform gyrus; POT = posterior occipitotemporal area; SMG = supramarginal gyrus; AG = angular gyrus; SA = semantic aphasia; WA = Wernicke’s aphasia; PF+ = prefrontal areas; TP-only = temporoparietal only.

^a^Lesion size was estimated by overlaying a standardized grid of squares onto each patient’s template to determine the percentage of squares damaged relative to the complete undamaged template. Anterior superior temporal gyrus (aSTG) was obtained by assessing BA 22 on the fourth and fifth slice of the Damasio template: any damage in front of the midpoint was defined as lesioned anterior superior temporal gyrus. Posterior superior temporal gyrus (pSTG) was restricted to the back half of the superior temporal gyrus, using the fifth and sixth slide of the Damasio template. A scan for Patient PG was unavailable; a radiographer’s report identified frontal and capsular damage.

The two patient groups were further subdivided into those with damage restricted to temporoparietal regions (the ‘TP-only’ group) and those with lesions encroaching into prefrontal areas (the ‘PF+’ group), including left inferior frontal gyrus (BA 44 and BA 45). Four cases with Wernicke’s aphasia had damage restricted to posterior temporal regions and four had damage extending anteriorly to frontal regions. There were eight semantic aphasia cases with damage extending to prefrontal regions and five with damage restricted to posterior temporal regions (Table 2).

### Neuropsychological assessment

#### Background neuropsychology

General neuropsychological testing included digit span (Wechsler, 1987), Ravens Coloured Progressive Matrices test of non-verbal reasoning (Raven, 1962), the Visual Object and Space Perception battery (VOSP, Warrington and James, 1991), Elevator Counting with and without distraction from the Test of Everyday Attention (Robertson *et al.*, 1994), and the Brixton Spatial Rule Attainment task (Burgess and Shallice, 1997). The patients were also examined on a standard battery of semantic tests to assess their comprehension of pictures, environmental sounds and words (presented simultaneously in spoken and written forms unless otherwise stated). These included: (i) Pyramids and Palm Trees (Howard and Patterson, 1992), a two alternative-forced-choice test of semantic associations for pictures and words; (ii) three components of the Cambridge 64-item semantic test battery (Bozeat *et al.*, 2000): spoken word–picture matching using 10 semantically-related response options, plus picture and word versions of the Camel and Cactus Test (a four alternative-forced-choice test tapping semantic associations); (iii) a three alternative-forced-choice test 96-item synonym judgement task (Jefferies *et al.*, 2009); and (iv) an environmental sounds battery, which involved matching environmental sounds-to-pictures, spoken words-to-pictures, written words-to-pictures and sounds-to-written words (Bozeat *et al.*, 2000). There were 10 semantically-related response options.

#### Cyclical matching tasks

**Figure 2:**
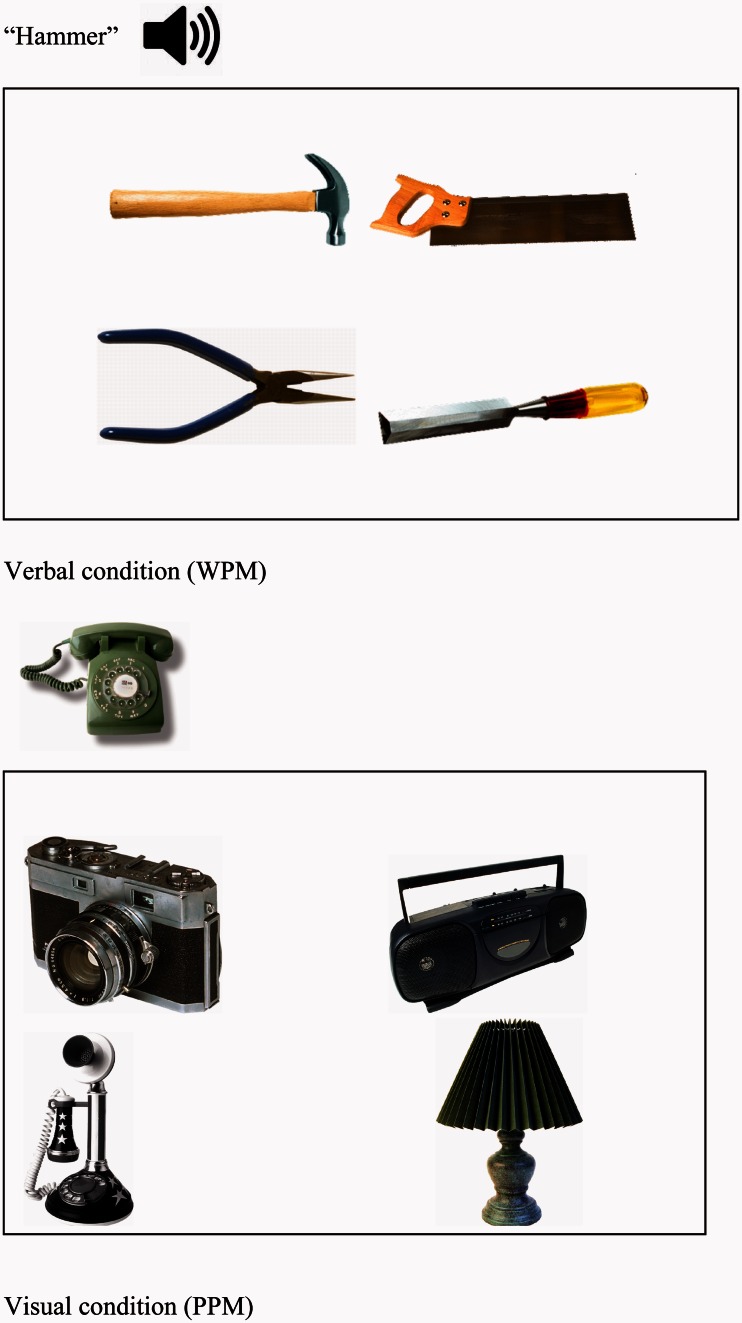
**Examples of trials used in the cyclical item matching task.** PPM = Picture-Picture matching; WPM = Word-Picture matching.

These picture–picture matching and word–picture matching tasks were used previously by Gardner *et al.* (2012, Experiment 1) and Warrington and Crutch (2004). Participants selected one of four pictures matching a spoken word or picture probe (Fig. 2). Items were presented repeatedly such that the target on one trial became the distractor on another, until all items within a semantic category had been tested. This completed one cycle. There were four cycles for each set of items, which probed the items in the set in a pseudorandom order, followed by a short break. This removed the potential confound between time and cycle, as cycle four of set one was presented before cycle one of set two. Participants had 10 s to point to the target, and immediately after each response the researcher triggered the next trial. There were four practice items before the start of each block. The same probes were presented as pictures and words. Testing was carried out in four blocks using an ABBA design to control for order effects across modalities. The stimuli consisted of 40 inanimate objects. These were grouped into 10 semantic sets (tools, electrical items, drink containers, clothes, household appliances, kitchen tools × 2, furniture × 2 and vehicles). The experiment was run using E-prime 1.1

## Results

### Background neuropsychology

#### Non-semantic tasks

Results are provided in Table 3. There was a significant difference between groups in digit span [*t*(17) = 2.538, *P = *0.021]. Patients with Wernicke’s aphasia were at floor on this task, whereas only 7/13 semantic aphasia cases were below the normal cut-off. There were no differences between patients with semantic aphasia and Wernicke’s aphasia on the Ravens Coloured Progressive Matrices test of non-verbal reasoning (*t* < 1), Brixton (*t* < 1), the Elevator Counting task with or without distraction (*t* < 1), or any subscale of the Visual Object and Space Perception (VOSP) battery [*t*(13) ≤ 1.447, *P ≥ *0.172]. Patients with semantic aphasia and and those with Wernicke’s aphasia showed evidence of impaired executive control. Some individuals in both groups were also impaired on visual tasks. Dot counting from VOSP battery is influenced by the ability to produce number words, whereas cube analysis draws on executive skills, making these scores hard to interpret. Moreover, spatial deficits are unlikely to explain the effects of cycle on our experimental tasks. Although visual search is relevant for global performance in this paradigm, visual impairment should have a relatively stable impact across trials. In addition, many of the patients with semantic aphasia reported here were previously shown to (i) have unchanging performance across cycles for blocks of semantically unrelated items in the same paradigm; and (ii) declining picture naming performance across cycles—even though visual search requirements are substantially reduced in this task (Jefferies *et al.*, 2007).

**Table 3:** Background performance: non-semantic tasks

Patient	Group	Digit span	RCPM	VOSP				TEA		Brixton
				Dot counting	Position discrimination	Number location	Cube analysis	No distraction	Distraction	
Max			36	10	20	10	10	7	10	54
Control mean (SD)		7 (0)^a^	32.6 (2.3)^a^	9.9 (0.3)	19.6 (0.9)	9.4 (1.1)	9.2 (1.2)	6.6 (1.2)	8.2 (2.8)	30 (4.8)^a^
Normal cut-off		5	28^b^	9.5	17.8	7.2	6.8	4.2	2.6	28
HN	SA – TP-only	6	20*	8*	19	9	4*	7	9	28
SC	SA – TP-only	6	22*	10	17*	10	9	7	1*	25*
ME	SA – TP-only	6	13*	3*	15*	2*	4*	7	9	11*
KS	SA – TP-only	8	31	NT	NT	NT	NT	5	9	28
EW	SA – TP-only	4*	30	10	20	10	7	NT	NT	33
PG	SA – PF+	6	23*	5*	20	9	10	3*	0*	26*
NY	SA – PF+	3*	26*	10	20	10	5*	3*	2*	34
BB	SA – PF+	5	24*	10	18	8	2*	0*	4	23*
DB	SA – PF+	4*	31	6*	0*	10	3*	2*	2*	31
GH	SA – PF+	2*	32	10	4*	0*	0*	6	1*	18*
EC	SA – PF+	0*	12*	3*	14*	10	6*	1*	1*	24*
KA	SA – PF+	0*	12*	0*	14*	6*	0*	5	5	6*
LS	SA – PF+	4*	6*	6*	16*	8	4*	2*	3	14*
EL	WA – TP-only	2*	27*	7*	20	10	6*	0*	0*	25*
MR	WA – TP-only	2*	31	9*	19	5*	6*	7	2*	16*
CW	WA – TP-only	4*	29	10	19	6*	10	7	7	39
DMC	WA – TP-only	1*	23*	NT	NT	NT	NT	NT	NT	NT
DR	WA – PF+	1*	10*	NT	NT	NT	NT	NT	NT	NT
LaS	WA – PF+	1*	21*	NT	NT	NT	NT	NT	NT	NT
DL	WA – PF+	NT	22*	NT	NT	NT	NT	NT	NT	NT
CB	WA – PF+	2*	25*	NT	NT	NT	NT	NT	NT	NT

*Denotes impaired performance. Control performance and normal cut-offs taken from the following published texts except where stated.

^a^Norms from 15 healthy controls tested at the University of York, average age 68, four male.

^b^2 SD below mean of controls tested at the University of York.

RCPM = Raven’s Coloured Progressive Matrices (Raven, 1962); VOSP = Visual Object and Space Perception battery (Warrington and James, 1991) section 5–8; TEA = Test of Everyday Attention (Robertson *et al.*, 1994); BSRA = Brixton Spatial Rule Attainment Task (Burgess and Shallice, 1997); NT = not tested; WA = Wernicke’s aphasia; SA = semantic aphasia; TP = temporoparietal; PF = prefrontal.

#### Semantic tasks

Results are displayed in Table 4. All patients showed some degree of impairment in both modalities (written word or picture), except one patient with Wernicke’s aphasia who did not show a deficit for pictures (Patient CW).

**Table 4:** Background performance: semantic tasks

Patient	Group	Spoken WPM	Naming	CCTp	CCTw	PPTp	PPTw	Synonyms	Environmental sounds test			
									Written word- picture	Spoken word- picture	Sound- picture	Sound- written word
Max		64	64	64	64	52	52	96	48	48	48	48
Control mean (s.d.)		63.7 (0.5)	62.3 (1.6)	58.9 (3.1)	60.7 (2.06)	51.2 (1.4)	51.1 (1.1)	94.4 (1.2)	NT^a^	47.8 (0.6)	41.2 (2.5)	40.8 (3.8)
Normal cut off		63	59	53	57	49	49	92	NT	46.6	36.2	33.2
HN	SA – TP-only	50***	51***	54	54*	35***	44***	89**	42***	16***	36	NT
SC	SA – TP-only	59***	28***	47**	56	29***	39***	71***	48	41***	32**	32
ME	SA – TP-only	50***	5***	13***	33***	29***	39***	80***	40***	40***	33**	35
KS	SA – TP-only	46***	21***	44***	NT	NT	NT	81***	NT	NT	NT	NT
EW	SA – TP-only	57***	45***	45**	48***	50	52	86***	38***	45*	22***	NT
PG	SA – PF+	58***	46***	44***	40***	42***	43***	69***	44***	47	33**	25**
NY	SA – PF+	60***	55**	36***	39***	47*	42***	69***	47	40***	28***	34
BB	SA – PF+	53***	10***	38***	30***	41***	35***	63***	26***	33***	26***	27**
DB	SA – PF+	46***	39***	51*	46***	NT	NT	54***	38***	36***	21***	NT
GH	SA – PF+	60***	19***	45**	29***	NT	NT	71***	NT	NT	NT	NT
EC	SA – PF+	40***	1***	32***	20***	NT	NT	41***	NT	NT	NT	NT
KA	SA – PF+	35***	0***	46**	36***	44***	44***	60***	36***	21***	22***	14***
LS	SA – PF+	48***	5***	15***	16***	31***	39***	47***	33***	35***	27***	17***
EL	WA – TP-only	30***	24***	49*	36***	48	36***	62***	45**	21***	30**	24**
MR	WA – TP-only	32***	11***	45**	46***	50	39***	66***	40***	26***	20***	17***
CW	WA – TP-only	51***	41***	55	55*	51	52	89**	47	30***	21***	22***
DMC	WA – TP-only	16***	0***	NT	NT	42***	39***	NT	NT	NT	NT	NT
DR	WA – PF+	9***	3***	NT	NT	47*	33***	NT	NT	NT	NT	NT
LaS	WA – PF+	32***	1***	NT	NT	46**	34***	NT	NT	NT	NT	NT
DL	WA – PF+	8***	2***	NT	NT	46**	32***	NT	NT	NT	NT	NT
CB	WA – PF+	30***	0***	NT	NT	42***	43***	NT	NT	NT	NT	NT

*Denotes impaired performance. * ≤ 0.05, ** ≤ 0.01, *** ≤ 0.001 using a modified *t*-statistic to examine whether an individual is significantly below a control group, taking into account control group size, mean and standard deviation (Crawford *et al.*, 2010). Control performance and normal cut-offs taken from the following published texts except where stated.

^a^Norms for analysis taken from spoken word-picture matching using the same stimuli. Spoken Word-Picture Matching (WPM) from the Cambridge Semantic Battery (Bozeat *et al.*, 2000); Synonym judgment (Jefferies *et al.*, 2009); Environmental Sounds Test (Bozeat *et al.*, 2003); CCT = Camel and Cactus task in picture and written word forms (Bozeat *et al.*, 2000); PPT = Pyramids and Palm Trees task in picture and written word forms (Howard and Patterson, 1992); NT = not tested; WA = Wernicke’s aphasia; SA = semantic aphasia; TP = temporoparietal; PF = prefrontal.

On the Pyramids and Palm Trees test, repeated-measures ANOVA revealed no main effect of group [*F*(1,14) = 2.858, *P = *0.113] or modality [*F*(1,15) = 2.246, *P = *0.155] but an interaction between group and modality [*F*(1,15) = 13.247, *P = *0.002]. Wernicke’s aphasia patients were less impaired than those with semantic aphasia on picture trials, but the two groups showed similar performance for words.

The 64 item word-picture matching task showed a significant group difference between semantic aphasia and Wernicke’s aphasia [*t*(18) = 4.895, *P < *0.001] with higher performance in patients with semantic aphasia. This identity matching task arguably has fewer semantic control demands than association matching tasks like Pyramids and Palm Trees, as there is no requirement to apply conceptual knowledge in a flexible fashion to determine the relevant relationship in each trial. However, word–picture matching is highly dependent on the ability to decode the spoken probe word and access its meaning, which is a core deficit in Wernicke’s aphasia.

The other semantic tasks were examined in fewer patients (10–16 out of 21 in total). The Camel and Cactus test showed no effect of group [*F*(1,11) = 1.202, *P = *0.296] or interaction of modality and group (*F* < 1). There was no significant group effect in any condition of the environmental sounds task [*t*(11) ≤ 1.607, *P ≥ *0.136] or in the synonym test (*t* < 1). The synonym judgement task additionally allowed us to assess the effect of frequency on comprehension (Supplementary material), as the absence of frequency effects is another hallmark of semantic access impairment (Warrington and Shallice, 1979; Warrington and Cipolotti, 1996; Hoffman *et al.*, 2011*a*; Almaghyuli *et al.*, 2012). Of three patients with Wernicke’s aphasia tested, none showed a significant frequency effect, whereas in the semantic aphasia group, just 2 of 13 patients showed a difference (Supplementary Table 3). Furthermore, in one of these cases, performance was significantly greater for low frequency items, potentially reflecting the greater control demands for high frequency words which have greater contextual diversity (Hoffman *et al.*, 2011*a*, *b*). In contrast, other patients with ‘storage’ rather than ‘access’ semantic deficits show robust comprehension benefits for high frequency items on the same task (Jefferies *et al.*, 2009).

#### Cyclical matching tasks

Healthy controls show ceiling level performance on the cyclical matching tasks and, if anything, a slight speeding up of reaction time with repetition (Gardner *et al.*, 2012). Therefore, the analyses focused only on the patient data.

##### Omnibus ANOVA

**Figure 3:**
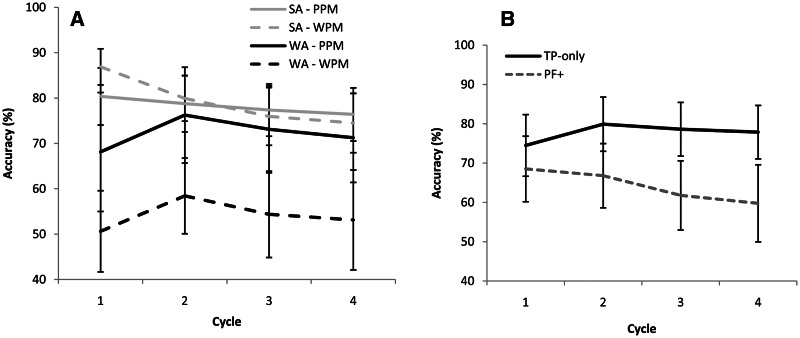
**Accuracy (%) across cycles.** (**A**) Data from semantic aphasia (SA) and Wernicke’s aphasia (WA) patients for word-picture matching (WPM) and picture-picture matching (PPM). (**B**) Prefrontal (PF+) and temporoparietal (TP-only) patients, including cases from both Wernicke’s aphasia and semantic aphasia groups and combining word and picture modalities. Error bars show standard error of mean.

Patients with semantic aphasia and Wernicke’s aphasia showed different effects of cycle and modality (Fig. 3A). A 2 × 4 × 2 repeated-measures ANOVA of modality (picture–picture matching or word–picture matching), cycle, and aphasia group (semantic aphasia or Wernicke’s aphasia) revealed a significant main effect of cycle [*F*(3,17) = 4.355, *P = *0.019], which interacted with aphasia group [*F*(3,17) = 7.250, *P = *0.002] (see below). There was also a marginally significant main effect of aphasia group [*F*(1,19) = 4.080, *P = *0.058] a main effect of modality reflecting poorer performance for words [*F*(1,19) = 6.858, *P = *0.017] and an interaction of aphasia group and modality [*F*(1,19) = 6.298, *P = *0.021] (see below). There were no other significant interactions. As noted in the Supplementary material, these effects of aphasia classification are likely to reflect the distribution of temporal lobe damage in the two groups.

##### Cycle

Patients with Wernicke’s aphasia and semantic aphasia showed the same decline in accuracy from cycle 2 to 4, but patients with Wernicke’s aphasia showed initial improvement between cycles 1 and 2, whereas semantic aphasia cases showed a decline. Independent samples *t*-tests (averaging across both modalities) revealed a significant aphasia group difference at cycle 1 [Bonferroni *t*(19) = 3.802, *P = *0.004] but no differences at other cycles [*t*(19) ≤ 1.360, *P ≥ *0.1]. Indeed, when rerunning the ANOVA without cycle 1, there was no longer an interaction of cycle and aphasia group (*F* < 1) or a main effect of aphasia group [*F*(1,19) = 2.173, *P = *0.157] although there was an effect of cycle [*F*(2,18) = 5.696, *P = *0.012].

##### Modality

The interaction of modality and aphasia group reflected equivalent performance for the two groups on the picture–picture matching task, whereas the Wernicke’s aphasia cases showed poorer accuracy on word–picture matching. Independent samples *t*-tests (averaged across all cycles within each modality) found no significant difference between patients with semantic aphasia and Wernicke’s aphasia on picture–picture matching (*t* < 1). However, there was a significant difference between aphasia groups for word–picture matching [Bonferroni *t*(19) = 2.914, *P = *0.02]. The ANOVA revealed no interaction of cycle and modality, and no three-way interaction between cycle, aphasia group and modality (*F* < 1), indicating that patients with Wernicke’s aphasia were consistently worse at the word–picture matching task than patients with semantic aphasia across cycles.

#### Left inferior frontal gyrus damage

The effect of left inferior frontal gyrus damage is shown in Fig. 3B. Repeated-measures ANOVA examined the following factors: left inferior frontal gyrus damage (TP-only or PF+ groups), modality (word–picture matching versus picture–picture matching), aphasia group (semantic aphasia, Wernicke’s aphasia) and cycle (1–4). Only main effects and interactions reflecting the presence or absence of left inferior frontal gyrus damage are reported here, since the other factors were considered above. There was a near-significant main effect of left inferior frontal gyrus damage [*F*(1,17) = 3.975, *P = *0.062] suggesting that PF+ patients may have been slightly more impaired than TP-only patients overall. PF+ patients had a larger lesion volume [*t*(18) = 2.822, *P* = 0.011], but this did not appear to explain the differential effects of cycle in PF+ and TP-only groups. There was no correlation between lesion size and the maximal change between cycles (expressed as a single variable in each case) for either picture–picture matching: r = 0.270, *P* = 0.250, or word–picture matching: r = 0.304, *P* = 0.193. This suggests that lesion location, rather than size, predicts semantic access deficits. There was an interaction of cycle and left inferior frontal gyrus damage [*F*(3,15) = 4.930, *P = *0.014]; PF+ patients showed a greater drop in accuracy across cycles than those with damage restricted to TP-only regions. Between-subjects *t*-tests comparing (i) cycles 1 to 4; and (ii) cycles 2 to 4 were computed for PF+ and TP-only patient groups (averaged across modalities). PF+ patients showed a decline in accuracy between cycles 1 and 4 [Bonferroni *t*(11) = 3.621, *P = *0.008] and cycles 2 and 4 [Bonferroni *t*(11) = 3.911, *P = *0.004]. TP-only patients did not show a difference between cycles 1 and 4 [*t*(8) = 1.484, *P* > 0.1] or cycles 2 and 4 [*t*(8) = 1.178, *P* > 0.2]. There were no other interactions involving the status of left inferior frontal gyrus, suggesting that both groups and modalities contributed to these effects of lesion location (although this analysis lacks statistical power to detect a subtle interaction with aphasia classification).

Analysis at the level of individual patients largely converged with these findings (Supplementary material). Patients with Wernicke’s aphasia showed consistent modality effects (pictures > words), whereas semantic aphasia cases did not. Significant effects of cycle were found in five semantic aphasia PF+ patients and effects approached significance in a further two cases (i.e. declining accuracy was seen in 7/8 individuals). Effects of cycle were not found in any of the TP-only patients from either group. This analysis also failed to detect effect of cycle in individual PF+ Wernicke’s aphasia cases (Supplementary Table 4).

#### Consistency across cycles and modality

A characteristic symptom of ‘access’ impairment is inconsistent retrieval of the same items when probed repeatedly (Warrington and McCarthy, 1983; Warrington and Crutch, 2004). Jefferies and Lambon Ralph (2006) found inconsistency in semantic aphasia cases across tasks with differing executive demands, and consistent performance when demands were broadly equivalent, such as when the items were presented in two modalities within the same task. We used logistic regression to assess accuracy consistency across cycles (which might have differing control demands) and between modalities (which should have similar control demands). To achieve this, we used performance on earlier cycles (cycles 1, 2 and 3) to predict scores on later cycles (cycles 2, 3 and 4, respectively), while including the additional variables of modality, lesion location, aphasia group, word frequency and individual patient ID in the model. We also used performance on picture–picture matching to predict accuracy on the word–picture matching task, including cycle and the other variables in the model.

##### Modality

Both Wernicke’s aphasia and semantic aphasia cases showed significant consistency across modalities. Word–picture matching accuracy was significantly predicted by picture–picture matching accuracy (W = 12.229, *P < *0.001). There were also effects of lesion (W = 9.621, *P = *0.002) aphasia group (W = 25.658, *P < *0.001) and patient ID (W = 281.375, *P < *0.001). There were no other significant factors, and no significant interactions. The reverse contrast (predicting picture–picture matching from word–picture matching) yielded similar results, but the main effect of aphasia group became non-significant.

##### Cycle

Item-by-item accuracy on cycles 2, 3, 4 was significantly predicted by performance on the same items in the previous cycle (1, 2 and 3, respectively) (W = 115.320, *P < *0.001). There were also significant effects of modality (W = 25.684, *P < *0.001) and patient ID (W = 290.510, *P < *0.001). There were interactions between accuracy on the previous cycle and aphasia group (Wernicke’s aphasia versus semantic aphasia) (W = 45.628, *P < *0.001) plus accuracy on the previous cycle and lesion location (PF+ versus TP-only) (W = 4.437, *P = *0.035). The three-way interaction term (cycle, lesion location and aphasia group) was not significant when this was added to the model (W = 2.161, *P = *0.142). By examining the same analyses for subgroups of patients in each modality separately, we were able to interpret these interactions as follows: semantic aphasia PF+ patients showed no consistency across sets of trials hypothesized to have varying control demands (i.e. between earlier and later cycles), whereas semantic aphasia TP-only patients showed some evidence of consistency across cycles on the visual task (not the verbal task). Wernicke’s aphasia cases showed significant levels of consistency, and this was again most evident on the visual task, with the Wernicke’s aphasia TP-only group showing the most consistent performance on this task. This is displayed in Supplementary Table 5.

##### Error analysis

A full error analysis is described in the Supplementary material. First, we analysed the proportion of errors that were perseverations and omissions across cycles (Supplementary material). Perseverations went up across cycles in both groups, but omissions were greatest at cycle 1 for the patients with Wernicke’s aphasia (and not for the semantic aphasia PF+ group). This might have reflected the initial difficulties that patients with Wernicke’s aphasia had in accessing semantics from inputs: this deficit might be ameliorated by repetition (Supplementary Table 6). We also examined whether errors for a particular target were consistent semantic confusions (i.e. the same incorrect response option was chosen across trials; Supplementary material). A deficit of semantic control was predicted to create consistent errors, since items which are most similar compete for selection (e.g. knife and fork). This prediction was confirmed by our results, displayed in Supplementary Table 7.

## Discussion

Although semantic aphasia and Wernicke’s aphasia have a long-standing history of study (Head, 1926; Eggert, 1977), they have rarely been directly compared. Both disorders are considered to reflect an ‘access’ disorder rather than an impairment/degradation of semantic representations *per se* (Warrington and Cipolotti, 1996; Jefferies and Lambon Ralph, 2006). Yet the term ‘access disorder’ can refer to two different deficits, either impaired entry into semantics from a particular modality (e.g. spoken words), or deficient control over the retrieval of semantic information. This study was able to answer key clinical and theoretical questions with regard to semantic ‘access’ disorders, including: (i) which type of ‘access’ disorder is present in patients with Wernicke’s aphasia versus patients with semantic aphasia; (ii) whether ‘access’ disorders are limited to the verbal domain; and (iii) how the distribution of temporal and frontal lobe damage relates to access impairments. This was achieved using verbal and non-verbal versions of the cyclical matching task. Whilst this assessment has become paradigmatic of ‘access’ semantic disorders, it is typically investigated in cases specifically selected to show declining comprehension with cycle. In contrast, we examined performance on this task systematically and comparatively in case series of Wernicke’s aphasia and semantic aphasia patients.

There were clear differences in the nature of the comprehension impairment in semantic aphasia and Wernicke’s aphasia, which reflected the distribution of temporal lobe damage in these groups. Patients with Wernicke’s aphasia showed poorer comprehension of spoken words than pictures, and input processing deficits characterized by omission errors that were initially ameliorated when stimuli were repeated, following greater damage to anterior-to-mid superior temporal gyrus. In contrast, patients with semantic aphasia showed equivalent impairment of verbal and non-verbal tasks and no beneficial effects of repetition, coupled with greater damage to occipital-temporal cortex. Nevertheless, the Wernicke’s aphasia and semantic aphasia groups showed a similar decline in verbal and non-verbal matching performance as competition increased in later cycles, and in both groups, this decline in performance with repetition was associated with damage to left inferior frontal gyrus. This poorer comprehension on later cycles reflected difficulty inhibiting previous targets, resulting in more perseverations of the preceding response. Our findings confirm a dual-deficit in Wernicke’s aphasia, i.e. deficient auditory/phonological input-processing plus impaired control over conceptual activation affecting both word and picture tasks (Robson *et al.*, 2012). This is compatible with the idea that key parts of the semantic network (both for representation and control) are multimodal (Jefferies and Lambon Ralph, 2006; Binder *et al.*, 2009; Lambon Ralph, 2014).

The semantic aphasia and Wernicke’s aphasia cases also showed other classic features of access impairment (Warrington and Shallice, 1979), including (i) weak or absent frequency effects in a synonym judgement task; and (ii) inconsistent performance when the same items were probed repeatedly. The first of these findings is thought to reflect the higher ‘contextual diversity’ of frequent words: high frequency items occur in a wider range of contexts and are therefore more easily associated with the distracter words in a synonym judgement task, increasing control demands (Hoffman *et al.*, 2011*a*; Almaghyuli *et al.*, 2012). The consistency analyses also appeared to reflect fluctuations in control demands. Both groups showed consistent performance across modalities, as the executive demands of word and picture trials were broadly matched (Jefferies and Lambon Ralph, 2006). Patients with semantic aphasia showed more inconsistency than Wernicke’s aphasia cases across cycles, consistent with their executive-semantic deficits in the absence of input processing deficits.

The largest difference between Wernicke’s aphasia and semantic aphasia came with the first repetition of the items: Wernicke’s aphasia showed an increase in performance, where patients with semantic aphasia showed no such facilitation. Moreover, patients with Wernicke’s aphasia frequently ask for repetition of spoken input in everyday settings and formal assessments. Presumably, repetition of the stimulus boosts initial poor encoding and patients utilize this powerful effect across many different situations. Similarly, patients with Wernicke’s aphasia show repetition priming effects, absent in some other types of aphasia (Blumstein *et al.*, 2000). These effects have been linked to deficits in activating lexical representations and in auditory working memory (Janse, 2008). For the first time, however, our findings indicate that this repetition priming effect goes beyond the verbal domain: patients with Wernicke’s aphasia found it difficult to activate the meaning of an item from a single presentation irrespective of modality. In contrast, patients with semantic aphasia performed at their best on the first cycle: they were able to maximize access to semantic information with the initial input. Cortical regions showing reduced activity with repetition (e.g. a semantic priming or adaptation effect)—including anterior parts of left inferior frontal gyrus and posterior middle temporal gyrus (Wagner *et al.*, 1997; Gold *et al.*, 2006; Badre and Wagner, 2007)—are more often intact in patients with Wernicke’s aphasia than in those with semantic aphasia. This may explain why patients with Wernicke’s aphasia receive benefit from repetition when semantic aphasia patients do not.

Over and above aphasia type, damage to the left inferior frontal gyrus was shown to predict negative effects of cycle in word and picture matching tasks. A similar conclusion was drawn in parallel studies of patients with prefrontal versus posterior temporal glioma (Campanella *et al.*, 2009, 2012). Patients with left prefrontal cortex lesions have difficulty overcoming competition from previously-relevant responses and adapting their semantic processing when the target changes (Corbett *et al.*, 2008; Jefferies *et al.*, 2008). This function is increasingly required in cyclical semantic matching tasks, as the distracter on one trial becomes the target on the next and thus the target must be identified from a field of previously selected and highly active items (Jefferies *et al.*, 2007). Once initial difficulties in activating concepts from inputs have been overcome, the semantic access impairments that occur in both semantic aphasia and Wernicke’s aphasia are best explained in terms of damage to this modulatory system in prefrontal cortex, as opposed to abnormalities within the representational system *per se*.

Previous studies have suggested that damage to left inferior frontal gyrus specifically disrupts lexical selection during speech production (Damian *et al.*, 2001; Maess *et al.*, 2002; Belke *et al.*, 2005; Moss *et al.*, 2005; Schnur *et al.*, 2006, 2009; Hsiao *et al.*, 2009; Robinson *et al.*, 2010). This study provides strong evidence that this competition-related deficit is not specific to speech production or even to verbal semantic processing: instead, damage to the left inferior frontal gyrus produces parallel problems in word and picture comprehension tasks, indicating that the control system supports the retrieval and selection of amodal concepts as opposed to word forms alone (Gardner *et al.*, 2012). There is already evidence that the left inferior frontal gyrus responds to non-verbal as well as verbal semantic tasks (Wagner *et al.*, 1997; Chee *et al.*, 2000; Bright *et al.*, 2004; Visser *et al.*, 2012; Krieger-Redwood *et al.*, 2015) and this region is engaged by a multitude of different semantic tasks with high executive demands (Noonan *et al.*, 2013). In particular, the mid-to-posterior part of the left inferior frontal gyrus (damaged in both semantic aphasia and Wernicke’s aphasia patients) is crucial for overcoming ‘post-retrieval selection’, i.e. interference from activated representations or responses that are no longer relevant (Thompson-Schill *et al.*, 1997; Badre *et al.*, 2005).

Beyond the left inferior frontal gyrus, semantic control is associated with a distributed network including left posterior middle/inferior temporal cortex, intraparietal sulcus, pre-supplementary motor area and right inferior frontal gyrus. Some of these regions support domain-general executive control, such as the inferior frontal sulcus and intraparietal sulcus (Duncan, 2010; Woolgar *et al.*, 2011). Others appear to be more specifically semantic in their function, notably left anterior inferior frontal gyrus and posterior middle temporal gyrus (Noonan *et al.*, 2013). Both left inferior frontal gyrus and posterior middle temporal gyrus show stronger activation in functional MRI studies when semantic control demands are maximal (Thompson-Schill *et al.*, 1997; Badre *et al.*, 2005; Whitney *et al.*, 2011*a*; Noonan *et al.*, 2013), and transcranial magnetic stimulation to these regions elicits equivalent disruption of high-control tasks (Whitney *et al.*, 2011*b*). Moreover, patients with semantic aphasia with left prefrontal and temporoparietal-only lesions show equivalent effects of various semantic control manipulations, including ambiguity, probe-target connectedness and distractor strength (Noonan *et al.*, 2010). This distinction between cyclical matching tasks (which specifically implicate left inferior frontal gyrus in control; Schnur *et al.*, 2006, 2009; Campanella *et al.*, 2009; Gardner *et al.*, 2012) and other situations suggests that although left inferior frontal gyrus and posterior temporal areas jointly support semantic control, they may make different contributions: the left inferior frontal gyrus might be important when the goals for semantic retrieval change and previous responses are no longer relevant, whereas both structures might work together to determine the correct response when relatively automatic stimulus-driven semantic retrieval is insufficient to support understanding.

In summary, the unique contribution of this study is to show that ‘refractory’ effects (i.e. negative effects of cycle) are comparable for words and pictures, not only in patients with semantic aphasia (selected to have multimodal semantic impairment) but also in patients with Wernicke’s aphasia (selected to show poor single-word comprehension and speech punctuated with phonological or neologistic errors). These effects are linked to damage to left prefrontal cortex (left inferior frontal gyrus), which has been previously associated with the control of competition from previously relevant responses. We conclude that patients with Wernicke’s aphasia have two types of semantic access impairment—both difficulty with initial conceptual activation (ameliorated by repetition) and difficulty in the face of strong competition (increased by repetition), whereas patients with semantic aphasia show the second type of semantic access deficit in isolation.

## Supplementary Material

Supplementary material
