# Supplementary material for: Varieties of semantic ‘access’ deficit in Wernicke’s aphasia and semantic aphasia
Source: Brain. 2015 Oct 10;138(12):3776–92. doi: 10.1093/brain/awv281 (PMC4655340; doi:10.1093/brain/awv281)
Supplement: Supplementary material [file 3ba53103c7cb3d79cbb815f82818c260_brain-2014-00932-File006.pdf]

Supplementary materials for Thompson et al. "Varieties of semantic access impairment"

Supplementary Analysis 1: Separating groups of patients on the basis of temporal lobe lesion location

Aphasia classification was related to the distribution of damage in the temporal lobe: SA patients had greater involvement of occipital-temporal areas while WA had more damage to STG/SMG. However, the lesions of the two groups were still partially overlapping in these regions. We reproduced the key effects of aphasia classification in a subset of patients selected to have distinct temporal lobe lesions by excluding four cases (EL from the WA group and BB, GH, and EC from the SA group). In this subset, all of the WA cases and none of the SA cases had damage to mid-to-anterior STG. The key findings from the cyclical matching tasks (Supplementary Table 1) and the background neuropsychological measures (Supplementary Table 2) were reproduced in this subset of patients, supporting the view that effects of aphasia classification can be related to temporal lobe lesion location.

Supplementary Table 1: Cyclical matching data for groups separated by temporal lobe lesion location

	**F**	**p**
**Cycle**	10.416	.002*
**Cycle*aphasia group**	12.681	.001*
**Cycle*lesion**	8.265	.004*
**Modality**	3.081	.103
**Modality*aphasia group**	5.599	.034*
**Modality*lesion**	.379	.549
**Modality*cycle**	.571	.645
**Modality*aphasia group*lesion**	.269	.613
**Cycle*aphasia group*lesion**	2.579	.107
**Modality*cycle*aphasia group**	.278	.840
**Modality*cycle*lesion**	.099	.959
**Modality*cycle*aphasia group*lesion**	3.174	.067

Note: The table reports the comparison of WA and SA patients with distinct temporal lobe lesions. Cycle = Cycles 1 to 4. Aphasia group = semantic aphasia (SA) vs. Wernicke's aphasia (WA). Lesion = with and without damage to prefrontal cortex. Modality = word-picture matching vs. picture-picture matching.

Supplementary Table 2: Background neuropsychological scores for groups separated by temporal lobe lesion location

	N	t statistic	p
Semantic			
PPTw	15	1.313	.212
PPTp	15	2.310	.038*
PPT: modality by group interaction	15	6.204[a]	.014*
Spoken WPM	17	4.509	< .001*
CCTw	11	1.053	.320
CCTp	12	.990	.346
Synonym judgment	12	.632	.542
Sounds – WW-P	11	.722	.488
Sounds – SW-P	11	.985	.350
Sounds – S-P	11	1.867	.095
Sounds – S-WW	8	.977	.366
Non-semantic			
RCPM	17	.406	.690
VOSP	11	.762	.466
RSRA	12	.475	.645
TEA – no distraction	11	1.559	.154
TEA – with distraction	11	.019	.985
Digit span	17	3.352	.004*

Note: The table reports the comparison of WA and SA patients with distinct temporal lobe lesions. N = number of patients in each analysis. [a] = F statistic. * = group difference to p < .05. PPT = Pyramids and Palm Trees task of semantic associations, presented as pictures and words (Howard & Patterson, 1992). Spoken WPM = word-picture matching from Cambridge Semantic Battery (Bozeat et al., 2000). CCT = Camel and Cactus task presented as pictures and words (Bozeat et al., 2000). Synonym judgment (Jefferies et al., 2009). Sounds = Environmental Sounds Test (Bozeat et al., 2003), with written word-picture matching (WW-P), spoken word-picture matching (SW-P), sound-picture matching (S-P) and sound-written word matching (S-WW) versions. RCPM = Raven's Coloured Progressive Matrices (Raven; 1962); VOSP (Visual Object and Space Perception battery; Warrington & James, 1991) subtests 5-8; BSRA = Brixton Spatial Rule Attainment Task (Burgess & Shallice, 1997); TEA (Test of Everyday Attention; Robertson et al., 1994).

Supplementary Analysis 2: Effects of word frequency on synonym judgement

Patients with semantic access impairment fail to show benefits of word frequency in comprehension tasks, unlike those with semantic dementia (Warrington & Cipolotti, 1996; Jefferies & Lambon Ralph, 2006; Jefferies, et al., 2009). Patients degraded semantic knowledge show relative preservation of concepts that are commonly encountered, while those with access disorders show a weakening of this effect (or in some cases even a reversal): this might reflect the higher 'contextual diversity' of high frequency words, which increases their control demands (Hoffman, et al., 2011; Almaghyuli, et al., 2012). Frequent words appear in a wider range of contexts and thus have a variety of possible interpretations – moreover, in the synonym judgement task we used, the distracters were from the same frequency range as the probe and target, and thus participants with deficient executive-semantic control might have found it more difficult to avoid spurious associations between the probe and distracters (e.g., in the trial MONEY with CASH, CAR or CHURCH, the synonym is CASH but an association between MONEY and BUYING A CAR or COLLECTING MONEY AT CHURCH might be retrieved erroneously). To assess whether the SA and WA cases showed another key characteristic of semantic access disorder, we examined the influence of word frequency on comprehension in this task. Data were available for all 13 SA cases but only 3 WA cases. The results are shown in Supplementary Table 3.

Supplementary Table 3: Analysis of frequency effects in synonym judgement

**Group**	**Patient**	**HF (%)**	**LF (%)**	Frequency effect (p value)	Comparison to SD patients (p value)
**SA – TP-only**	HN	97.9	89.6	.089	
**SA – TP-only**	SC	75.0	72.9		.091
**SA – TP-only**	ME	87.5	79.2		
**SA – TP-only**	KS	91.7	77.1	.016	
**SA - TP-only**	EW	79.2	79.2		.081
**SA – PF+**	PG	68.8	75.0		.032
**SA – PF+**	NY	68.8	75.0		.032
**SA – PF+**	BB	66.7	64.6		.075
**SA – PF+**	DB	60.4	52.1		
**SA – PF+**	GH	64.6	83.3	.049	.008
**SA – PF+**	EC	41.7	43.8		.026
**SA – PF+**	KA	64.6	60.4		.089
**SA – PF+**	LS	52.1	45.8		.082
**WA – TP-only**	EL	64.6	64.6		.057
**WA – TP-only**	MR	75.0	62.5		
**WA – TP-only**	CW	91.7	93.8		.087

HF = high frequency, LF = low frequency. Data displayed as percentage accuracy. P values displayed are $p < .1$. The difference between scores on high vs. low frequency trials was analysed. 'Frequency effect' column reflects logistic regressions run on each individual with frequency and imageability in the model, frequency p values reported. An RSDT analysis (Crawford & Garthwaite, 2005) was also used to compare WA/SA patients with a group of SD patients. Significant p values in this comparison reflect no frequency effects or reverse frequency effects in WA/SA, in comparison to large frequency effects in SD.

Supplementary Analysis 3: Individual performance on cyclical matching tasks

We performed logistic regression to assess the extent to which different variables predicted accuracy in individual patients. An initial omnibus model including all the patients reproduced the effects reported in the main analysis using ANOVA: we found a significant predictive effect of aphasia group ($W = 18.131$, $p < .001$), modality ($W = 70.486$, $p < .001$), lesion location (PFC vs. TP-only; $W = 11.078$, $p = .001$), and patient ID ($W = 440.877$, $p < .001$). We then added in three interactive terms, and found a significant interaction of cycle and lesion location ($W = 7.510$, $p = .006$), lesion location and modality ($W = 5.117$, $p = .024$), and aphasia group by modality ($W = 96.630$, $p < .001$). There was no significant three-way interaction (between aphasia group, lesion location and cycle) when this term was added to the model ($W < 1$). Since individual patient ID was a strong predictor of performance in this model, we performed further logistic regressions to assess patients' performance individually, shown in Supplementary Table 4. We would not expect every individual case to show a significant influence of cycle using this approach, since group-level analyses have greater statistical power to detect subtle effects and our patients were *not* specifically selected to show this pattern, unlike many previous studies (Warrington & McCarthy, 1983; Warrington & Cipolotti, 1996; Crutch & Warrington, 2008). Nevertheless, analysis of individual patient data confirmed main of the key effects at the group level including strong modality effects (pictures > words) in WA but not SA, and effects of cycle that were significant or approaching significance in nearly all PF+ SA patients. Effects of cycle were not found in any of the TP-only patients, from either group. This analysis also failed to detect effect of cycle in individual PF+ WA cases. While null results for this subset of patients might reflect insufficient power to detect subtle effects in single cases, we cannot rule out the possibility that the combination of PFC and ventral posterior temporal damage in SA increases the effects of cycle in this group. Occipital-temporal areas show co-activation with PFC during executively demanding tasks (Duncan, 2010) and therefore SA cases might typically have damage to two distinct components supporting executive control.

Supplementary Table 4: Logistic Regression examining individual patients' performance

Group	Lesion	Patient	Modality	Cycle
SA	TP-only	HN	.007, W > P	n.s.
SA	TP-only	SC	.083, W > P	n.s.
SA	TP-only	ME	n.s.	n.s.
SA	TP-only	KS	.003, W > P	n.s.
SA	TP-only	EW	n.s.	n.s.
SA	PF+	PG	n.s.	.063
SA	PF+	NY	.001, W > P	.032
SA	PF+	BB	n.s.	n.s.
SA	PF+	DB	n.s.	< .001
SA	PF+	GH	< .001, P > W	.011
SA	PF+	EC	n.s.	.089
SA	PF+	KA	< .001, P > W	.001
SA	PF+	LS	n.s.	.003
WA	TP-only	EL	< .001, P > W	n.s.
WA	TP-only	MR	< .001, P > W	n.s.
WA	TP-only	CW	< .001, P> W	n.s.
WA	TP-only	DMC	< .001, P > W	n.s.
WA	PF+	DR	< .001, P > W	n.s.
WA	PF+	LaS	.047, P > W	n.s.
WA	PF+	DL	< .001, P > W	n.s.
WA	PF+	CB	.008, P > W	n.s.

Variables included in the model: cycle, modality. Table displays p values at < .1. n.s. = not significant (p > .1.). W > P = higher performance on words than pictures, P > W = higher performance on pictures than words.

Supplementary Table 5: Logistic Regression of consistency in each subgroup

**Task**	**Subgroup**	**Previous accuracy (Wald)**	**p value**
**WPM**	SA PF+	.415	n.s.
	SA TP-only	1.281	n.s.
	WA PF+	9.355	.002
	WA TP-only	8.101	.004
**PPM**	SA PF+	0	n.s.
	SA TP-only	8.694	.003
	WA PF+	48.012	< .001
	WA TP-only	70.477	< .001

Variables included in the model: previous cycle accuracy, patient ID, word frequency. Table displays p values at < .05.

Supplementary Analysis 4: Error analysis in cyclical matching tasks

As well as accuracy, patterns of errors can inform our understanding of patients' deficits. We coded errors as perseverations (which occurred when participants selected the same item as on the immediately preceding trial), omissions (when no response was made) or 'other' (i.e., another incorrect response was selected but not the one chosen on the previous trial). All the following analyses used logistic regression to examine the effects of aphasia group, patient ID, modality, cycle, lesion location and cycle by aphasia group interaction. We used data from all 13 SA patients and 5 WA patients (3 WA data sets were unavailable for analysis at this level – MR, DMC and DR). Data is shown in Supplementary Table 6.

Perseverations: As a proportion of errors, both SA and WA patients showed an increase in perseverations over cycles: there were more perseverations on cycle 4 than cycle 1: Wald = 8.838, $p = .003$. This increase in perseverations is consistent with increasing competition from activated representations on later cycles. The effect of cycle did not interact with aphasia group, or lesion location and there were no other main effects.

Omissions: In contrast to perseverations, omissions were more likely on cycle 1 than cycle 4: Wald = 29.829, $p < .001$. This drop was more dramatic for WA patients than SA, and WA patients tended to show more omissions overall. Consequently, there was a main effect of aphasia group: Wald = 8.384, $p = .004$, and an interaction of aphasia group with cycle: Wald = 8.985, $p = .003$. The data below suggest that the SA TP-only patients showed a similar decline in omissions to WA patients, but lesion location was not a statistically significant predictor of omissions. This statistical outcome is consistent with the suggestion that WA patients had initial difficulties accessing semantics from inputs, and that this deficit was ameliorated with repetition.

Supplementary Table 6: Percentage of error types per subgroup across cycles

		Error type (%)	Cycle			
			1	2	3	4
**WPM**	WA PF+	Perseveration	22.8	35.8	30.9	48
		Omission	19.3	0	1.8	0
		Other	57.9	64.2	67.3	52
	WA TP-only	Perseveration	14.8	31.6	28.6	22.7
		Omission	11.1	0	4.8	4.5
		Other	74.1	68.4	66.7	72.7
	SA PF+	Perseveration	14.8	31.6	28.6	22.7
		Omission	9.1	6.5	8.2	12.2
		Other	61.8	59.1	52.7	53.4
	SA TP-only	Perseveration	11.8	24	20.7	20
		Omission	17.6	0	6.9	0
		Other	70.6	76	72.4	80
**PPM**	WA PF+	Perseveration	6.7	25	23.1	31
		Omission	66.7	28.1	20.5	11.9
		Other	26.7	46.9	56.4	57.1
	WA TP-only	Perseveration	28.6	16.7	16.7	42.9
		Omission	0	0	0	0
		Other	71.4	83.3	83.3	57.1
	SA PF+	Perseveration	21.3	37.7	29.6	36.8
		Omission	29.5	23.4	16.3	14.9
		Other	49.2	39	54.1	48.3
	SA TP-only	Perseveration	12.2	36.8	26.5	46.3
		Omission	24.4	15.8	14.7	2.4
		Other	63.4	47.4	58.8	51.2

WPM = word-picture matching, PPM = picture-picture matching.

Supplementary Analysis 5: Consistent semantic confusions

Finally, we assessed whether particular incorrect targets were reliably selected for individual probes (e.g., does 'cat' consistently get confused with 'dog', as compared with 'rabbit' or 'horse' within the same block?). A deficit of semantic control might create consistent errors of this nature, since some items within a set will be more semantically similar to the target than others, and therefore compete more strongly for selection. Results are shown in Supplementary Table 6. The analysis examined whether the identity of the target could be predicted from the item that was chosen on incorrect trials; in blocks showing significant results, there was an association between target identity and response identity, even though the wrong item was selected. On blocks where this interaction was not significant, different possible distracter items were chosen at random. There was a significant or marginally significant predictive value of probe identity for over half of the items. Furthermore, there was no further predictive value of all other variables, suggesting that patients, regardless of their group or lesion, behaved in a similar way.

Supplementary Table 7: Logistic regression of consistent semantic errors

Error type

		**Stimuli set 1**		
	**Bed**	**Bookcase**	**Desk**	**Stool**
**Bed**	-	8.57	12.82	26.92
**Bookcase**	19.05	-	76.92	38.46
**Desk**	47.62	45.71	-	34.62
**Stool**	33.33	45.71	10.26	-
**P value**		.003	< .001	
		**Stimuli set 2**		
	**Bottle**	**Bowl**	**Mug**	**Teapot**
**Bottle**	-	28.57	42.86	25.93
**Bowl**	63.64	-	28.57	29.63
**Mug**	22.73	33.33	-	44.44
**Teapot**	13.64	38.10	28.57	-
**P value**	.002			
		**Stimuli set 3**		
	**Bus**	**Car**	**Lorry**	**Motorbike**
**Bus**	-	22.22	38.89	30.77
**Car**	42.86	-	27.78	42.31
**Lorry**	47.62	38.89	-	26.92
**Motorbike**	9.52	38.89	33.33	-
**P value**	.036			
		**Stimuli set 4**		
	**Cabinet**	**Chair**	**Dresser**	**Sofa**
**Cabinet**	-	23.33	77.38	10.34
**Chair**	15.00	-	13.10	65.52
**Dresser**	77.50	20.00	-	24.14
**Sofa**	7.50	56.67	9.41	-
**P value**	< .001	.006	< .001	< .001
		**Stimuli set 5**		

	**Camera**	**Lamp**	**Telephone**	**Radio**
**Camera**	-	14.29	22.00	52.38
**Lamp**	11.76	-	64.00	14.29
**Telephone**	35.29	71.43	-	33.33
**Radio**	52.94	14.29	14.00	-
**P value**	.058	.057	< .001	.045
		**Stimuli set 6**		
	**Chisel**	**Hammer**	**Pliers**	**Saw**
**Chisel**	-	29.63	38.71	25.71
**Hammer**	31.82	-	29.03	45.71
**Pliers**	43.18	44.44	-	28.57
**Saw**	25.00	25.93	32.26	-
**P value**				
		**Stimuli set 7**		
	**Corkscrew**	**Jug**	**Kettle**	**Whisk**
**Corkscrew**	-	7.50	5.00	36.36
**Jug**	26.67	-	85.00	39.39
**Kettle**	0.00	65.00	-	24.24
**Whisk**	73.33	27.50	10.00	-
**P value**	.002	< .001	< .001	
		**Stimuli set 8**		
	**Fork**	**Knife**	**Spatula**	**Spoon**
**Fork**	-	34.15	13.04	24.49
**Knife**	45.83	-	65.22	24.49
**Spatula**	14.58	19.51	-	51.02
**Spoon**	39.58	46.34	21.74	-
**P value**	.005	.041	.001	.007
		**Stimuli set 9**		
	**Hoover**	**Iron**	**Oven**	**Toaster**
**Hoover**	-	43.48	40.00	30.43
**Iron**	72.22	-	20.00	30.43
**Oven**	16.67	30.43	-	39.13
**Toaster**	11.11	26.09	40.00	-
**P value**	.001			
		**Stimuli set 10**		
	**Jacket**	**Shirt**	**Shorts**	**Trousers**
**Jacket**	-	31.58	22.22	19.15
**Shirt**	40.00	-	33.33	25.53
**Shorts**	20.00	26.32	-	55.32
**Trousers**	40.00	42.11	44.44	-
**P value**	.056			.001

Logistic regression for each probe separately, to see if the target identity could be predicted from the error made. The DV was a binary coding of the target identity (e.g., either 'jacket' or 'not jacket'), and the IV was the item selected in error (e.g., shirt, shorts or trousers). P values show the significant predictive value of stimuli to $p < .1$. There were no other significant predictive variables (group, modality, lesion location or patient ID). Percentages show the proportion of each error attributable to the three possible stimuli within the set, each column equating to 100%. Chance level would be 33.3%.

References

Almaghyuli A, Thompson HE, Lambon Ralph MA, Jefferies E. Deficits of semantic control produce absent or reverse frequency effects in comprehension: Evidence from neuropsychology and dual task methodology. Neuropsychologia 2012; 50: 1968-1979.

Crutch SJ, Warrington EK. The influence of refractoriness upon comprehension of non-verbal auditory stimuli. Neurocase 2008; 14: 494 - 507

Hoffman P, Rogers TT, Lambon Ralph MA. Semantic diversity accounts for the “missing” word frequency effect in stroke aphasia: Insights using a novel method to quantify contextual variability in meaning. Journal of Cognitive Neuroscience 2011; 23: 2432-2446.

Jefferies E, Lambon Ralph MA. Semantic impairment in stroke aphasia versus semantic dementia: a case-series comparison. Brain 2006; 129: 2132-2147.

Jefferies E, Patterson K, Jones RW, Lambon Ralph MA. Comprehension of concrete and abstract words in semantic dementia. Neuropsychology 2009; 23: 492-499.

Warrington EK, McCarthy RA. Category specific access dysphasia. Brain 1983; 106: 859-878.

Warrington EK, Cipolotti L. Word comprehension - The distinction between refractory and storage impairments. Brain 1996; 119: 611-625.
