# Supplementary figures and images for: Varieties of semantic ‘access’ deficit in Wernicke’s aphasia and semantic aphasia
Source: Brain. 2015 Oct 10;138(12):3776–92. doi: 10.1093/brain/awv281 (PMC4655340; doi:10.1093/brain/awv281)

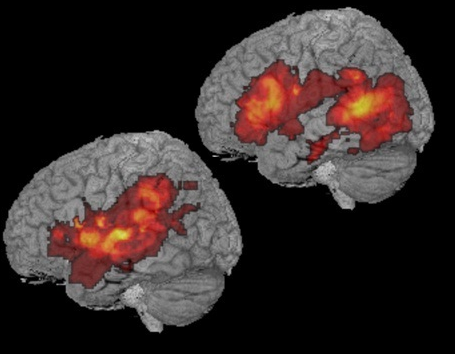

Supplement: Supplementary material [file 9f39b7520f9abb4460a7fedff09b44e2_brain-2014-00932-File007.png]
